# The Comparative Analysis of Single Plating Versus Double Plating in the Treatment of Unstable Bicondylar Proximal Tibial Plateau Fractures

**DOI:** 10.7759/cureus.46840

**Published:** 2023-10-11

**Authors:** Manish Raj, Santosh Kumar Singh, Ajay K Rajput, Simrat Pal Gill, Satyendra K Verma, Sushant S Sonarkar

**Affiliations:** 1 Department of Orthopedics, All India Institute of Medical Sciences (AIIMS), Deoghar, IND; 2 Department of Orthopedics, Maa Vindhyavasini Autonomous State Medical College, Mirzapur, IND; 3 Department of Orthopedics, Uttar Pradesh University of Medical Sciences (UPUMS), Saifai, IND; 4 Department of Orthopedics, Rajarshi Dashrath Autonomous State Medical College (RDASMC), Ayodhya, IND; 5 Department of Orthopedics, 7 Air Force Hospital, Kanpur, IND

**Keywords:** rasmussen's criteria, dual locking plate, radiological outcome, oxford knee score, schatzker, unstable bicondylar tibial plateau fracture

## Abstract

Introduction

In the present study, we aimed to compare the clinical and radiological results of the single lateral locking plate fixation method to the dual plate (DP) fixation method in cases of unstable bicondylar proximal tibial plateau fractures.

Materials and methods

Fifty-six patients managed surgically with internal fixation for unstable bicondylar tibial plateau fractures (UBTF) (Schatzker type V and type VI or Arbeitsgemeinschaft für Osteosynthesefragen/Orthopaedic Trauma Association {AO/OTA} type 41-C) over 36 months from January 2017 to December 2020 were included in this prospective study. All the fractures were fixed surgically either using dual locking plates through double incisions (DP group) or with a single lateral locking plate (single plate {SP} group). All intraoperative and postoperative complications were assessed and recorded. Oxford Knee Score (OKS), Rasmussen's functional grading system, and Rasmussen's radiological scoring system were used to evaluate the functional and radiological outcomes.

Result

All of the patients were followed for at least 12 months. Twenty-six patients were fixed with a single lateral locking plate, and 30 patients were fixed with a double-incision dual locking plate. The mean Oxford Knee Score (OKS) was 43.24 ± 4.46 in the DP group and 42.7 ± 2.57 in the SP group (P = 0.544). The mean Rasmussen's functional score (RFS) score in the present study was 26.6 ± 2.21 in the DP group and 24.97 ± 3.92 in the SP group (P = 0.056). At the final follow-up, the mean Rasmussen's radiological score (RRS) was 9.06 ± 1.01 in the DP group and 8.1 ± 0.81 in the SP group (P = 0.0003).

Conclusion

There are no statistically significant differences in the functional outcomes between the two groups, but higher benefits were found in the radiological outcomes in the dual plating group as compared to single lateral locking plate group.

## Introduction

Treating high-energy bicondylar tibial plateau fractures remains a challenge to orthopedic trauma surgeons because of their complicated intraarticular fracture patterns and the soft tissue insult associated with it [1,2]. Nearly 96% of tibial plateau fractures occur due to road traffic motorcycle accidents [3]. According to the Schatzker classification type V and type VI or according to the Arbeitsgemeinschaft für Osteosynthesefragen/Orthopaedic Trauma Association (AO/ATO) type 41-C, fractures are described as complex unstable bicondylar tibial plateau fractures (UBTF) that are associated with higher comminution and difficulty in the reconstruction of articular congruity and maintaining reduction [4]. Various surgical approaches and internal fixation techniques including single lateral locking plate and dual plating have been developed to treat these types of fractures, but the superior method is yet not clear and is debatable. While evaluating the literature, the long-term outcomes of both surgical fixation modalities show similar results, and there is no clear consensus on which fixation method leads to the best outcomes [5-8]. Studies on cadavers show that dual plate (DP) fixation had greater biomechanical strength and a lower rate of subsidence than a single lateral locking plate [9]. Various authors have advocated that stabilizing both medial and lateral columns with dual plate fixation through two incisions yields satisfactory functional outcomes in complex tibial plateau fractures [10-13]. However, limited soft tissue dissections are required with single plating as compared to dual plating to minimize the risk of postoperative wound complications [14]. A single lateral locking plate prevents the stripping of soft tissue for the additional medial plate with limited dissection. Percutaneous screw insertion through the guide arm in cases of a single fixed-angle locking plate applied laterally effectively engages the medial fragment to support the medial plateau and prevent varus collapse [15-17]. The failure of a single locking plate has been reported in cases of bicondylar tibial plateau fracture where there is a presence of medial articular fracture having a coronal component with a posteromedial fragment [17].

The search for the ideal implant (single plate {SP} versus dual plate) for internal fixation of these bicondylar tibial plateau fractures still continues. To help in resolving this controversy, we decided to compare the clinical and radiological results of the fixation using a single lateral locking plate and a dual locking plate in cases of unstable bicondylar tibial plateau fractures.

## Materials and methods

A prospective interventional randomized study was conducted over 36 months from January 2017 to December 2020 at a tertiary care center in Central India, after obtaining clearance from the Institutional Ethical Committee of Uttar Pradesh University of Medical Sciences (UPUMS/Dean/M/Ethical/121/2018). Written informed consent was obtained from all the participants.

An initial total of 60 patients having bicondylar tibial plateau fractures were included in this study. The inclusion criteria for the present study include skeletally matured patients above 18 years of age who presented with unstable bicondylar tibial plateau fracture (Schatzker type V and type VI or AO/ATO type 41-C) [18,19]. The exclusion criteria included Gustilo-Anderson open fracture type II and type III, pathological fractures, accompanying multiple fractures in the same limb, polytrauma, compartment syndrome, distal neurovascular compromise, and a follow-up shorter than one year.

All the patients included in the study were randomized according to a random number series using a computerized program. Randomization was done by an independent observer, and both the participants and the operating surgeons were unaware of the random number sequences. A single lateral locking plate was used in the SP group, which included 30 patients, while a dual locking plate was used in the DP group, which also included 30 patients. However, intraoperatively, we did not achieve the desired stability of both medial and lateral columns in four patients from a single plate group, and an additional medial plate was applied in these patients resulting in the exclusion of these patients from the present study. So, the final sample size for analysis was limited to 26 cases in the SP group and 30 cases in the DP group. To minimize bias, intention-to-treat (ITT) analysis was used for the analysis and result interpretation.

Preoperative management

At the time of admission, the patient's injured extremity was examined and clinically evaluated, with a particular focus on skin condition, swelling, joint effusion, neurovascular injury, and the shortening of the limb. To define the tibial plateau fracture geometry, standard anteroposterior (AP) and lateral radiographs were taken. In those cases where fracture patterns could not be properly assessed by radiographs, a CT scan with three-dimensional reconstruction was done. Schatzker and AO/ATO classifications were used to classify all tibia plateau fractures.

The condition of the skin and the surrounding tissues was the major determinant factor for the timing of fixation surgery for these fractures. Until the tissue edema settled (wrinkle sign appeared) and the skin condition became good enough to post the patient for surgery, calcaneal or lower tibial skeletal traction was applied to the patients with extreme swelling of soft tissue.

Surgery in both groups was performed by a team of four senior orthopedic surgeons from the same unit in the study hospital (each having experience of more than eight years). Using a pneumatic tourniquet and a folded pillow under the knee, all procedures were carried out, while the patient was supine and under spinal anesthesia.

Surgical technique for single lateral locking plate

The lateral condyle tibial fracture was reduced and fixed with a proximal tibial lateral locking compression plate in the group with a single lateral plate using the anterolateral approach. After proper soft tissue dissections, under C-arm fluoroscopy with longitudinal traction, joint line congruity was checked first. Percutaneous K-wires were used to hold the fracture fragments and maintain the reduction of the fracture fragments under the C-arm guidance. If there was articular depression, the depressed fragment was raised using a bone punch, and the empty space was filled with an autologous iliac crest bone graft.

Surgical technique for dual locking plate

Fracture fragments of the medial tibial condyle were fixed first. Depending upon the presence of a posteromedial fragment, an anteromedial or posteromedial incision was made extending distal to the pes anserinus. The proximal level of the incision was taken more posteriorly in the presence of the posteromedial fracture part. The pes anserinus was retracted anteriorly or posteriorly, and the medial tibial condyle was fixed with a 4.5 mm proximal tibial T or L buttress locking plate and screws, after contouring. The second lateral locking plate was applied in the same manner as described above.

A suction drain was used in all patients, and the wound was closed primarily. Third-generation cephalosporin (ceftriaxone) was given as an injectable antibiotic for seven days.

Postoperative management and evaluations

Under the direction of a physiotherapist, isometric quadriceps exercises with a passive movement of the knee joint were initiated on the second postoperative day, depending on the degree of fixation stability and patient compliance to pain. After 14 postoperative days, the sutures were taken out. In between physiotherapy sessions, patients were immobilized with a long knee brace. Depending on the stability of the fracture fixation and correlation with the X-ray, partial weight-bearing ambulation with a walker frame was started between 10 and 12 weeks after the fracture. Full weight-bearing walking was allowed only after the confirmation of the radiological union of the fracture on plain film. Bony union was defined radiographically when at least three cortices out of four united. Patients were called for follow-up biweekly for one month, then monthly for three months, and thereafter at six months and 12 months. At each follow-up visit, clinical and radiological assessment for fracture healing and alignment was done. Patients were examined for skin conditions including signs of infection, range of movement of the knee, implant failure, loss of reduction, or any other complication related to the union or soft tissue.

Functional and radiological outcomes were assessed with Rasmussen's functional score (RFS) [20], Rasmussen's radiological score (RRS) [21], Oxford Knee Score (OKS) [22], and range of motion of the knee joint.

**Figure 1:**
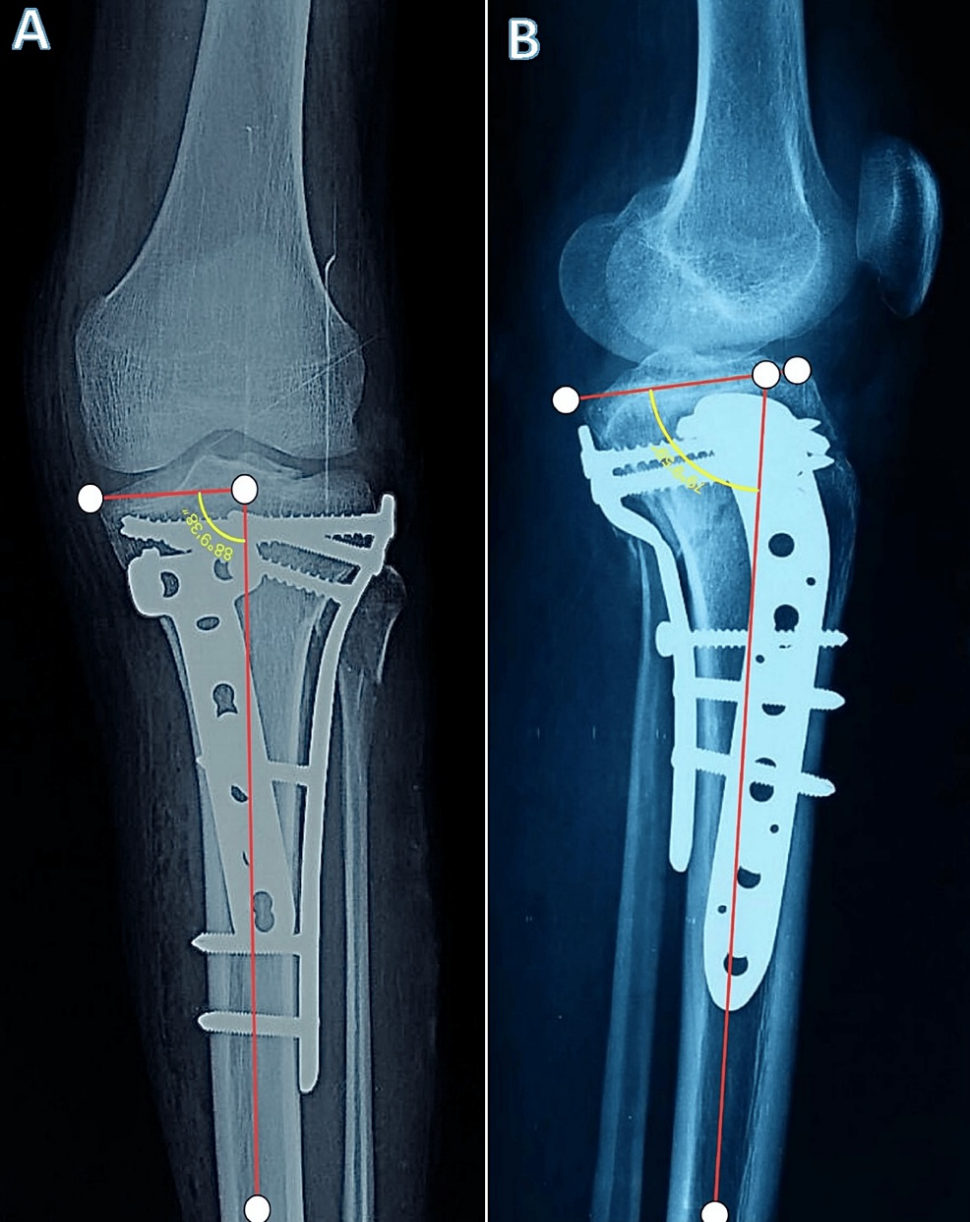
Measurement of the angles to evaluate the coronal and sagittal alignment of the fixation of the fracture of the proximal tibia managed by dual plating (lateral plate with posteromedial plate). (A) Measurement of the medial proximal tibial angle (MPTA). (B) Measurement of the posterior proximal tibial angle (PPTA).

**Figure 2:**
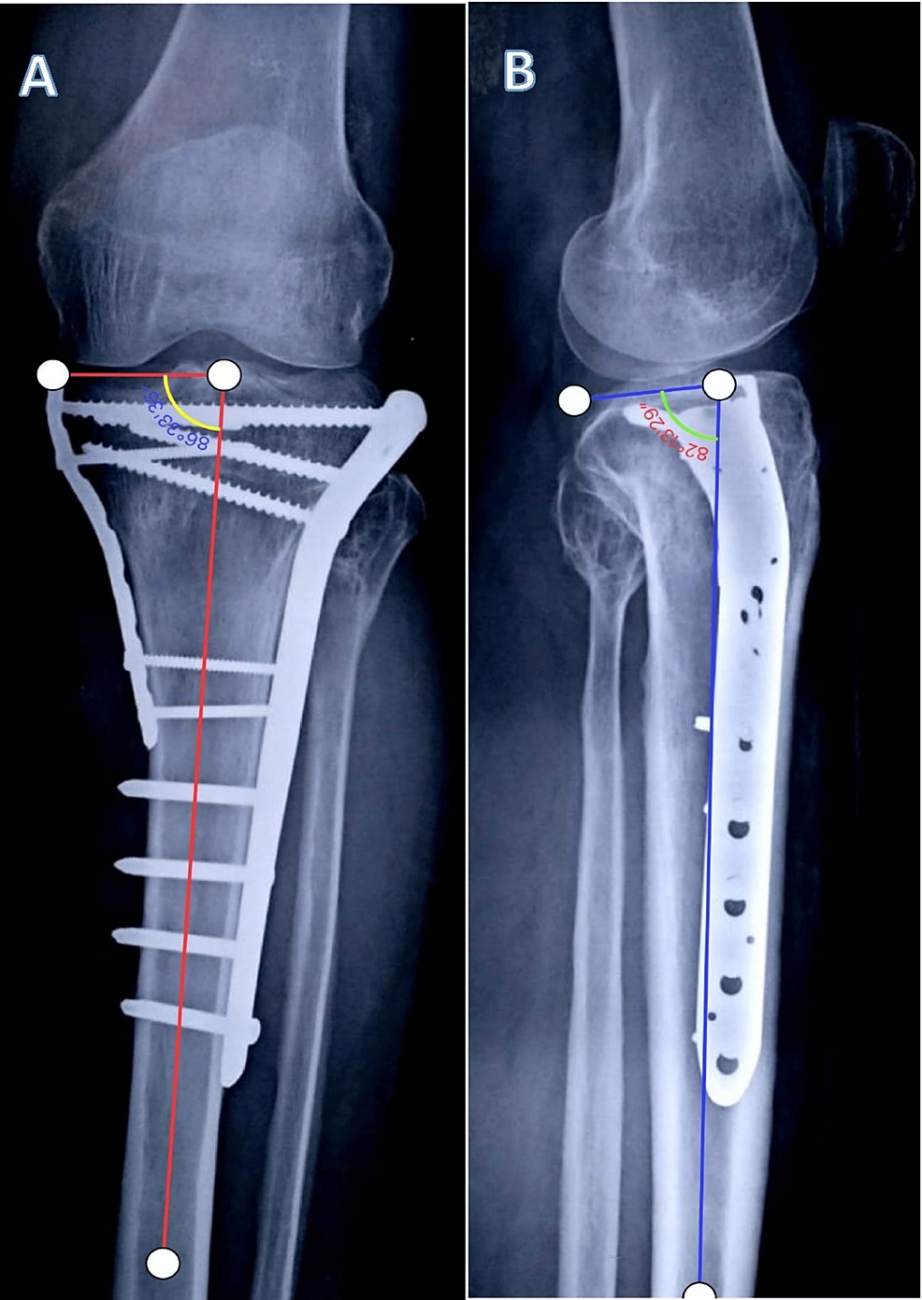
Measurement of the angles to evaluate the coronal and sagittal alignment of the fixation of the fracture of the proximal tibia managed by dual plating (lateral plate with medial plate). (A) Measurement of the medial proximal tibial angle (MPTA). (B) Measurement of the posterior proximal tibial angle (PPTA).

The postoperative radiological assessment of tibial plateau fracture reduction status and the alignment of the proximal tibia was done both in the sagittal and in the coronal plane through the posterior proximal tibial angle (PPTA) and medial proximal tibial angle (MPTA), respectively. PPTA was defined as the angle between the tangential line of the medial plateau and the perpendicular line of the anterior tibial cortex on lateral radiographs, while MPTA is the medial angle between the tangential line and anatomical axis of the tibia in AP radiographs as shown in Figure 1 and Figure 2.

Malalignment was defined as an MTPA ≥ 90°/MTPA ≤ 80° or PPTA ≥ 15°/PPTA ≤ 5°, while the secondary loss of alignment was defined as an increase of 3° malalignment when compared with the first postoperative radiograph [23]. Radiological assessment was done immediately after surgery until the last visit at the final follow-up.

Statistical analysis

The statistical analyses were performed using the Statistical Package for Social Sciences (SPSS) version 24.0 (IBM SPSS Statistics, Armonk, NY). Categorical variables were presented as numbers and percentage, while continuous variables were shown as the mean values ± standard deviation (SD) and range. Categorical variables between the two groups were compared by a chi-square test or Fisher's test, as appropriate. Continuous variables were compared by the independent samples t-test or Mann-Whitney U test, as appropriate. P < 0.05 was considered significant.

## Results

The demographic details of the patients of both groups were shown in Table 1.

**Table 1:** Demographic details of the patient. ^1^Data is shown as mean ± SD and N (%). P < 0.05 was considered significant. RTI, road traffic injury; AO/OTA, Arbeitsgemeinschaft für Osteosynthesefragen/Orthopaedic Trauma Association; SD, standard deviation

Serial number	Variables		Single lateral locking plate group (SP group), N (%)	Dual locking plate group (DP group), N (%)	P value
1	Age (years)^1^		41.31 ± 8.75	35.6 ± 13.8	0.07
2	Sex, male/female		20/6	26/4	0.34
3	Mode of injury	RTI	18 (69.23)	24 (80)	0.79
		Sport	1 (3.85)	1 (3.33)	
		Fall from standing	2 (7.69)	1 (3.33)	
		Fall from height	5 (19.23)	4 (13.33)	
4	Side involved	Right	14 (53.85)	11 (36.67)	0.19
		Left	12 (46.15)	19 (63.33)	
5	AO/OTA fracture type	41-C1	1 (3.85)	3 (10)	0.997
		41-C2	15 (57.69)	16 (53.33)	
		41-C3	10 (38.46)	11 (36.67)	
6	Schatzker fracture type	Type V	5 (19.23)	6 (20)	0.94
		Type VI	21 (80.77)	24 (80)	
7	Associated fracture		4 (15.38)	7 (23.33)	0.46
8	Alcohol use		3 (11.54)	4 (13.33)	1.00
9	Tobacco use		6 (23.08)	9 (30)	0.76
10	Diabetic		4 (15.38)	5	1.00

A final total of 56 patients having unstable bicondylar tibial plateau fractures (Schatzker type V and type VI or AO/ATO type 41-C) were included in both groups in this study. The mean follow-up time was 24 ± 2.1 months (range: 12-32 months) in the SP group and 22.4 ± 4.1 months (range: 12-36 months) in the DP group (P = 0.07). Among the demographic details, there were no significant differences found between the two groups regarding the mean age of the patient, sex, mode of injury, fracture side, fracture type, and associated fractures (P > 0.05). Intraoperative and postoperative details of the patients are depicted in Table 2.

**Table 2:** Intraoperative and postoperative variables of the patients. ^1^Data is shown as mean ± SD and N (%). P < 0.05 was considered significant. SD: standard deviation

Serial number	Variable		Single lateral locking plate group (SP group), N (%)	Dual locking plate group (DP group), N (%)	P value
1	Management	Primary	22 (84.62)	25 (83.33)	0.89
		Staged	4 (15.39)	5 (16.67)	
2	Preoperative stay (days)^1^		7.4 ± 4.1	8.9 ± 4.7	0.21
3	Duration of surgery (minutes)^1^		63.38 ± 26.65	79.43 ± 9.35	0.0031
4	Blood loss (mL)^1^		107.4 ± 51.6	146.6 ± 42.3	0.00008
5	Duration of hospital stay (days)^1^		10.73 ± 2.8	12.73 ± 4.40	0.05
6	Fluoroscopy time (number)^1^		27.33 ± 1.4	36.41 ± 5.1	0.021
7	Primary bone grafting		2 (7.69)	4 (13.33)	0.67
8	Duration of follow-up (months)^1^		24 ± 2.1	22.4 ± 4.1	0.07
9	Weight-bearing (weeks)^1^	Partial	14.7 ± 4.8	13.8 ± 3.07	0.40
		Complete	20.1 ± 3.28	18.95 ± 3.85	0.28
10	Fracture union (weeks)^1^		19.2 ± 3.5	18.4 ± 1.1	0.24

The mean duration between injury and definitive surgery was 7.4 ± 4.1 days in the SP group and 8.9 ± 4.7 days in the DP group (P = 0.21). There was a statistically significant difference in intraoperative parameters including the duration of surgery and blood loss during the surgery. The average duration of surgery in the DP group (79.43 ± 9.35 minutes) was higher as compared to the SP group (63.38 ± 26.65 minutes) (P = 0.0031). There was a statistically significant less blood loss (P = 0.00008) in the SP group (107.4 ± 51.6 mL) compared to the DP group (146.6 ± 42.3 mL). However, there was no difference between the groups regarding the duration of hospital stay (P = 0.05), partial weight-bearing (P = 0.4), and complete weight-bearing (P = 0.28). The average number of C-arm shots utilized during the procedure was used to estimate the amount of fluoroscopy time used. The number of shots used in the DP group (36.41 ± 5.1) was substantially greater (P = 0.021) than in the SP group (27.33 ± 1.4). Primary bone grafting was done in two patients of the SP group and four patients in the DP group (P = 0.67). No malalignment was found in both groups on the immediate postoperative radiograph. Bony union was achieved in all patients of both groups. The mean duration of the fracture union was 19.2 ± 3.5 weeks in the SP group, while it was 18.4 ± 1.1 weeks in the DP group (P = 0.24).

The postoperative functional score and radiological outcomes of the patients are shown in Table 3.

**Table 3:** Postoperative functional scores and radiological outcome of the patients. ^1^Data is shown as mean ± SD and N (%). P < 0.05 was considered significant. SD: standard deviation

Serial number	Variables		Single lateral locking plate group (SP group), N (%)	Dual locking plate group (DP group), N (%)	P value
1	Range of movement	≥120°	22 (84.62)	27 (90)	0.46
		≤120°	4 (15.39)	3 (10)	
2	Oxford Knee Score (OKS)^1^		42.7 ± 2.57	43.24 ± 4.46	0.544
3	Rasmussen's functional score (RFS)^1^		24.97 ± 3.92	26.6 ± 2.21	0.056
4	Medial proximal tibial angle (MPTA) (at the final follow-up)^1^		83.3 ± 7.32	87.1 ± 6.21	0.04
5	Postoperative proximal posterior tibial angle (PPTA) (at the final follow-up)^1^		12.4 ± 1.7	10.6 ± 1.9	0.0005
6	Rasmussen's radiological score (RRS)^1^		8.1 ± 0.81	9.06 ± 1.01	0.0003

The mean Rasmussen's radiological score (RRS) at the final follow-up was 9.06 ± 1.01 in the DP group and 8.1 ± 0.81 in the SP group, which is statistically significant (P = 0.0003). There was a significant difference between both groups concerning MPTA and PPTA at the final follow-up. The mean MPTA at the final follow-up was 87.10 ± 6.21 in the DP group and 83.30 ± 7.32 in the SP group (P = 0.04); the mean PPTA at the final follow-up was 10.60 ± 1.90 in the DP group and 12.40 ± 1.7 in the SP group (P = 0.0005). The functional outcome was compared using Rasmussen's functional score (RFS) and showed a nonsignificant difference between both groups (P = 0.544). The mean Oxford Knee Score (OKS) was 43.24 ± 4.46 in the DP group and 42.7 ± 2.57 in the SP group, which was statistically insignificant (P = 0.544).

Different kinds of complications were encountered in the present study, infection, articular depression, malalignment, secondary loss of reduction, failure of the implant, and knee stiffness, which are summarized in Table 4.

**Table 4:** Postoperative complications. The data has been presented as N (%). P < 0.05 was considered significant.

Serial number	Variables		Single lateral locking plate group (SP group), N (%)	Dual locking plate group (DP group), N (%)	P value
1	Infection	Superficial	1 (3.85)	3 (10)	0.54
		Deep	1 (3.85)	1 (3.33)	
2	Radiological malunion (at 12-month follow-up)	Articular depression	2 (7.69)	1 (3.33)	0.88
		Varus malalignment	4 (15.39)	1 (3.33)	
		Secondary loss of reduction	2 (7.69)	1 (3.33)	
		Failure of implant/loosening of screw	1 (3.85)	1 (3.33)	
3	Stiffness of knee joint		3 (11.54)	1 (3.33)	0.23

There was no significant difference in late complications between both groups (P = 0.88). Three patients (10%) in the DP group and a patient (3.3%) in the SP group had superficial infection postoperatively, which were managed with oral antibiotic therapy and local wound care. Deep infection was found in one patient in both groups who were treated with surgical debridement and intravenous antibiotic therapy. Infection was resolved in both cases. At the final follow-up, one patient in the DP group (3.33%) and four patients in the SP group (15.3%) were having varus malalignment. Secondary loss of reduction was depicted in follow-up radiographs in two patients of the SP group and one patient in the DP group. Implant failure was found in one patient in the SP group, for which another surgery was done for osteosynthesis, while one patient in the DP group had loosening of a screw, which was managed by the removal of that screw. Late complications include extension lag in the range of 0°-5° in three patients of the SP group, while one patient developed extension lag in the DP group. The average knee flexion was 123° in the DP group and 121° in the SP group. Knee stiffness was present in three patients in the SP group and one patient in the DP group.

**Figure 3:**
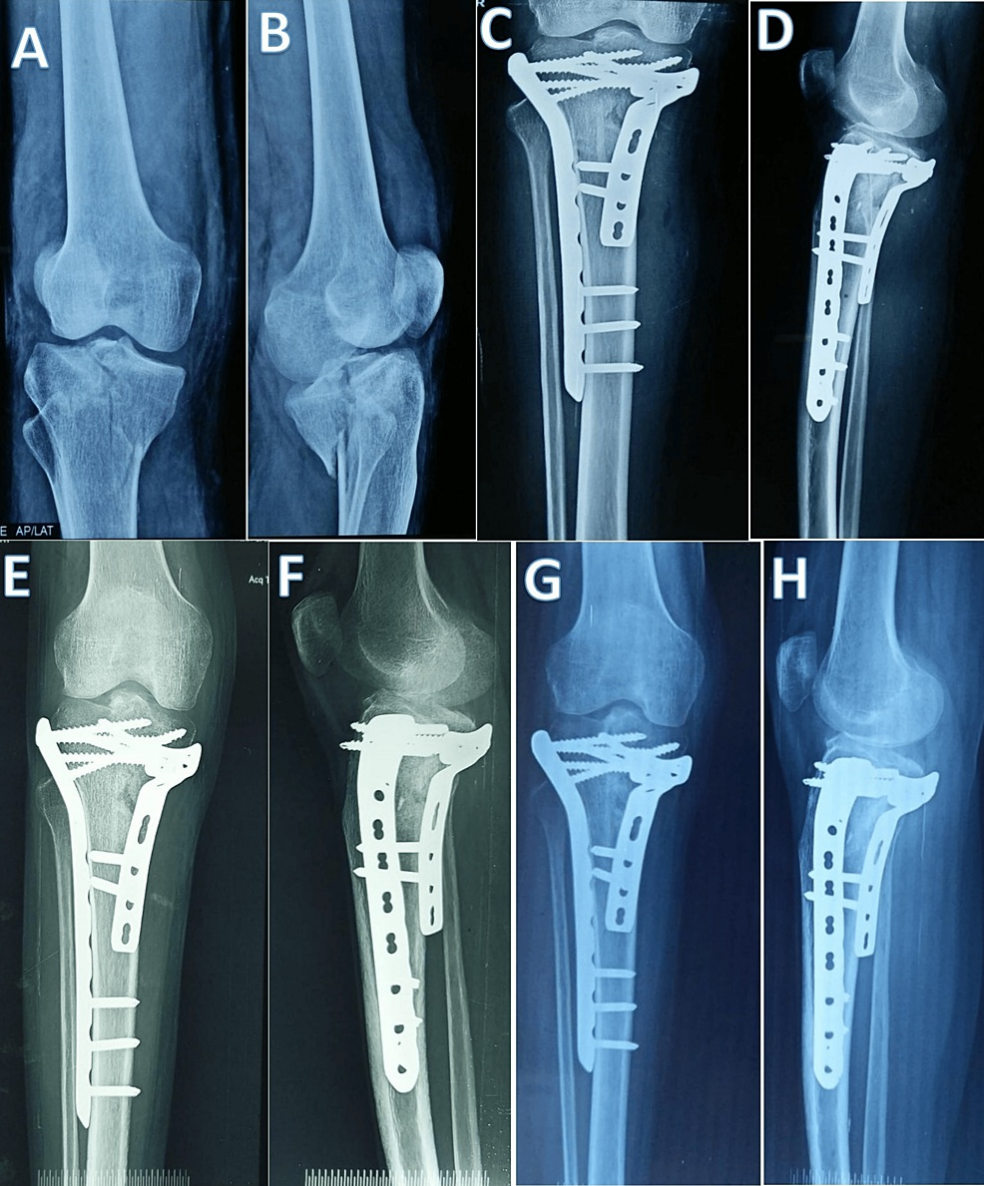
Follow-up radiographs of a 33-year-old male patient with a complex unstable right proximal tibial plateau fracture that was fixed with two plates. (A and B) Preoperative radiographs showing the proximal tibial plateau fracture pattern. (C and D) Immediate postoperative radiograph of the fracture fixed by dual plating (anterolateral plate and posteromedial plate). (E and F) Follow-up radiograph six months after surgery. (G and H) Follow-up radiograph 12 months after surgery.

The follow-up radiographs of a 33-year-old male patient who had an unstable bicondylar proximal tibial plateau fracture of the right side that was treated with dual plating (posteromedial plate and lateral plate) are shown in Figure 3.

**Figure 4:**
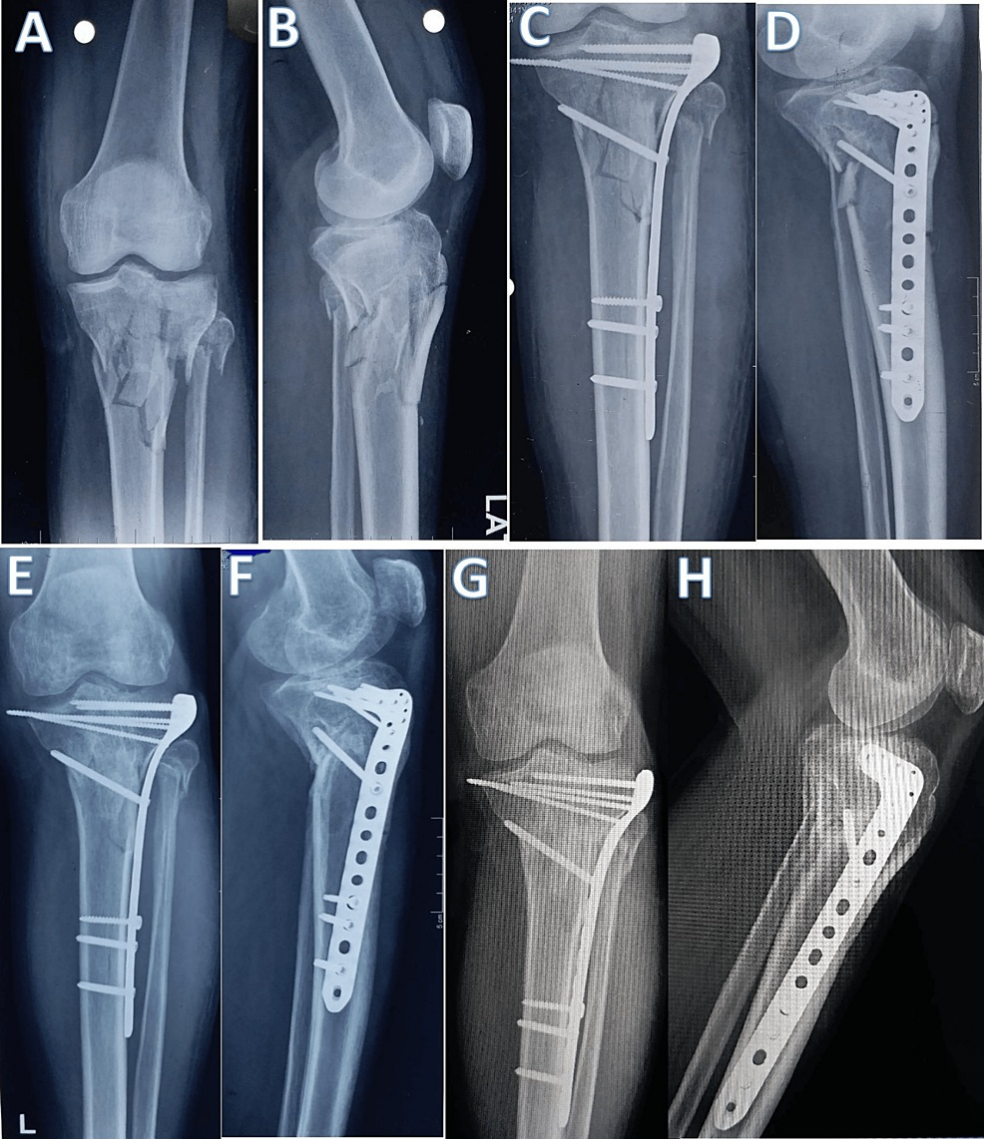
Follow-up radiographs of a 47-year-old male patient with a complex unstable left proximal tibial plateau fracture that was fixed with a lateral locking plate. (A and B) Preoperative radiographs showing the proximal tibial plateau fracture pattern. (C and D) Immediate postoperative radiograph of the fracture fixed by a single plate (lateral locking plate). (E and F) Follow-up radiograph six months after surgery. (G and H) Follow-up radiograph 12 months after surgery showing varus angulation.

The follow-up radiographs of a 47-year-old male patient who had an unstable bicondylar proximal tibial plateau fracture of the left side that was treated with a lateral locked plate are shown in Figure 4.

## Discussion

The internal fixation of high-energy unstable tibial plateau fractures is often subject to much debate within the literature. Dual plating (medial/posteromedial and lateral) or a single lateral locking plate has been advocated as a means of internal fixation in these cases [24-27]. However, there is no clear-cut consensus on the relative merits of double plate versus single plate in the literature [23,26,27]. A single lateral locking plate in cases of bicondylar tibial plateau fractures can support the medial plateau and prevent varus collapse with locking screws holding medial fragments. Limited soft tissue dissection and the submuscular application of a plate with the insertion of percutaneous screws through guide arms in cases of single plating help in the preservation of vascularity and soft tissue integrity by reducing additional further insult to the soft tissue [16,28,29]. This method reduces the intraoperative time effectively with better patient compliance [17].

In the present study, we found a statistically significant difference in the reduction of intraoperative surgical time and blood loss, in the single plate (SP) group as compared to the dual plate (DP) group. The difference in the duration of hospital stay was nonsignificant between both groups. Single plating does not, however, have the ability to fix every unstable bicondylar tibial plateau fracture and has its limitations. One or more of the following conditions, or a combination of them, have been reported in cases of tibial plateau fractures: medial intraarticular fracture line, small comminuted medial plateau fragment, medial articular fracture with a coronal component, and a posteromedial fragment [17]. Bicondylar tibial plateau fractures were reported to have a 28.8%-59% incidence rate of the posteromedial fragment [30]. When used laterally, one locking plate with a set angle might not be able to hold the posteromedial fragment effectively. The strength of the fixation may not be sufficient to counteract the displacement force, even if locking screws through the lateral plate hold the posteromedial fragment [17,18,28]. In cases of tibial plateau fractures with a coronal medial fracture line, the direction of locked screws is fixed and is mostly parallel rather than perpendicular to a coronal fracture line. High-energy bicondylar tibial plateau fractures involving both the medial and lateral columns (Schatzker type V/VI or AO/OTA types 41-C1, 41-C2, and 41-C3) require precise anatomical reduction and fixation of both columns to achieve the biomechanical strength. Dual plating stabilizes both the medial and lateral columns and provides superior biomechanical stability with stable fixation [7,9,26,31,32].

Studies on cadavers and biomechanics revealed that dual plate fixation was superior to a single lateral locking plate in terms of biomechanical strength and had a lower rate of secondary loss of reduction [23]. According to a biomechanical study on cadavers published by Higgins et al., dual plate fixation in bicondylar tibial plateau fractures results in less subsidence than single lateral plate fixation [9]. The outcomes were comparable to those of the biomechanical investigation by Mueller et al., which compared the stability of dual locking plate fixation to unilateral plate fixation in the treatment of unstable bicondylar tibial plateau fracture [32]. Yao et al. suggested that the shape of the medial condyle plays a significant role in the outcome of single versus dual plating [6]. Different authors in the past reported malalignment postoperatively. Neogi et al. reported a higher incidence of the malalignment of the proximal tibia during the subsequent follow-up in the single plate group (three cases, 10.9%) than in the dual plate group (two cases, 6.2%) [8]. In the present study, the malalignment of the proximal tibia was present in four cases (15.3%) in the single plate group and one case (3.33%) in the double plate group, with varus collapse being the most common reason for the postoperative change of the alignment of the proximal tibia. Malreduction on the immediate postoperative radiograph was found in two patients (7.6%) in the SP group and one patient (3.3%) in the DP group due to articular surface depression showing a step or gap of >2 mm. Various authors have reported better clinical and radiological outcomes in the management of complex proximal tibial plateau fractures with dual locking plates as compared to a single lateral locking plate in the past [28-30].

The mean Rasmussen's functional score (RFS) in the present study was 26.6 ± 2.21 in the dual plate group and 24.97 ± 3.92 in the single plate group, which was statistically nonsignificant (P = 0.056). The mean Oxford Knee Score (OKS) in the SP group was 42.7 ± 2.57, while it was 43.24 ± 4.46 in the DP group, which was also statistically nonsignificant (P = 0.5440). The mean Rasmussen's radiological score (RRS) at the final follow-up was 9.06 ± 1.01 in the DP group and 8.1 ± 0.81 in the SP group, which is statistically significant (P = 0.0003). There was a significant difference between both groups about MPTA and PPTA at the final follow-up. However, the loss of reduction and poor radiological final outcomes are also related to the inadequate fixation of the posterior component. Therefore, fixing with posterior incisions is advocated as necessary when medial-lateral incisions are insufficient in this region [25].

The limitation of fixation with dual plating includes extensive soft tissue dissections, which may increase the risk of wound complications. The incidence of deep wound infection in cases with dual plate fixation was reported to be 4.7%-8.4% by different authors [25-30]. In our study, there were three cases (10%) of superficial infection in the dual plate group and one case (3.8%) in the single plate group. Deep infection was present in one case (3.3%) in the DP group and one case (3.8%) in the SP group. All cases of superficial and deep infection in both groups were treated with prolonged antibiotic therapy and wound care. Soft tissue complications associated with dual plating can be minimized by gentle soft tissue handling and waiting for 5-6 days after an injury so that tissue edema would subside and skin condition would improve. Comparative analysis between the current study and other existing studies in the literature is shown in Table 5.

**Table 5:** A comparative analysis between the present study and other studies in the literature. SP, single plate; DP, double plate; CDP, classic dual plate; HDP, hybrid dual plate; KSS, Knee Society Score; RFS, Rasmussen's functional score; SMFA, Short Musculoskeletal Function Assessment; HSS, Hospital for Special Surgery; WOMAC, Western Ontario and McMaster Universities Osteoarthritis Index; OKS, Oxford Knee Score; NM, not mentioned; MPTA, medial proximal tibial angle; PPTA, posterior proximal tibial angle

Study	Type of study	Total number of patients		Mean age (years)		Mean follow-up duration (weeks)		Knee score		Wound complications		Malalignment		Secondary loss of reduction		Mean MPTA (at the final visit)		Mean PPTA (at the final visit)		Union rate (%)	
		SP	DP	SP	DP	SP	DP	SP	DP	SP	DP	SP	DP	SP	DP	SP	DP	SP	DP	SP	DP
Citak et al. (2019) [26]	Retrospective	10	10	51.2	51.3	27.8	24	72.9 (KSS); 22.9 (RFS)	79.1 (KSS); 24.3 (RFS)	0	1 (10%)	1	0	0	0	86.3	86.8	5.4	5.5	100%	100%
Patil et al. (2017) [24]	Prospective	18	19	46.5	42.4	24		30.38 (SMFA)	27.68 (SMFA)	0	1	2 (11%)	1 (5.6%)	2	1	83°	86°	NM	NM	100%	100%
Neogi et al. (2015) [8]	Prospective	29	32	41	37	22		79 (HSS)	80 (HSS)	2 (6.7%)	5 (15.6%)	17.24%	0%	2	1	NM	NM	NM	NM	100%	100%
Lee et al. (2013) [28]	Retrospective	15	19 (CDP); 11 (HDP)	49	53 (CDP); 51 (HDP)	18		36 (WOMAC)	34 (WOMAC); 32 (WOMAC)	3 (20%)	3 (15.7%) (CDP); 2 (18.1%) (HDP)	1	0	3	3 (CDP); 1 (HDP)	87.9	87.0 (CDP); 85.1 (HDP)	NM	NM	86.7%	79% (CDP); 91% (HDP)
Present study	Prospective	26	30	41.3	35.6	24	22.4	42.7 (OKS); 24.97 (RFS)	43.24 (OKS); 26.6 (RFS)	2	4	4	1	2	1	83.3	87.1	12.4	10.6	100%	100%

The main limitation of the present study was as follows: A small number of patients were studied, and there were a shorter duration of follow-up and associated soft tissue injuries such as ligamentous or meniscal damage, which were not discussed in our study but may influence the postoperative functional outcome. Due to the shorter follow-up period in our study, we may miss the post-traumatic knee arthritic changes, which occur with time. We suggest a large sample size of randomized controlled trials with a longer duration of follow-up to formulate a standard treatment protocol for the management of unstable high-energy bicondylar tibial plateau fractures.

## Conclusions

The results of this study show that there are no statistically significant differences between the functional outcomes of the dual locking plate group and the single lateral locking plate group for the treatment of unstable bicondylar proximal tibial plateau fractures. However, the dual plating groups had better radiological outcomes than the single plating groups, based on clinical follow-up evaluations.
